# ShinyCortex: Exploring Single-Cell Transcriptome Data From the Developing Human Cortex

**DOI:** 10.3389/fnins.2018.00315

**Published:** 2018-05-15

**Authors:** Jorge Kageyama, Damian Wollny, Barbara Treutlein, J. Gray Camp

**Affiliations:** ^1^Max Planck Institute for Evolutionary Anthropology, Leipzig, Germany; ^2^Max Planck Institute of Molecular Cell Biology and Genetics, Dresden, Germany; ^3^Department of Biosciences, Technical University Munich, Munich, Germany

**Keywords:** single cell RNA sequencing, cortex development, organoids, corticogenesis, single-cell transcriptomics

## Abstract

Single-cell mRNA sequencing (scRNA-seq) is a powerful method to identify and classify cell types and reconstruct differentiation trajectories within complex tissues, such as the developing human cortex. scRNA-seq data also enables the discovery of cell type-specific marker genes and genes that regulate developmental transitions. Here we provide a brief overview of how scRNA-seq has been shaping the study of human cortex development, and present ShinyCortex, a resource that brings together data from recent scRNA-seq studies of the developing cortex for further analysis. ShinyCortex is based in R and displays recently published scRNA-seq data from the human and mouse cortex in a comprehensible, dynamic and accessible way, suitable for data exploration by biologists.

New technologies to sequence the transcriptomes of single cells is having an enormous impact on developmental biology research (Tanay and Regev, 2017). This capacity to sequence single-cell transcriptomes allows the relatively unbiased analysis of diverse cell types within a complex tissue based on the abundance of messenger RNAs, an important component of a cell's state at any given time. Single-cell mRNA-seq (scRNA-seq) methods range from relatively low throughput, but high coverage across the full transcript (Picelli et al., 2013), to high throughput with coverage focusing on the 3' or 5' end of the transcript (Jaitin et al., 2014; Klein et al., 2015; Macosko et al., 2015), with most widely-used protocols selecting for poly-adenylated transcripts. Because scRNA-seq data are high-information content, new computational strategies have been developed to understand what the data represents. There are two general analytical methods that are typically used to analyze scRNA-seq data from developing tissues such as the cortex (Figure 1). First, cells can be clustered based on similar molecular signatures, which then allows for identification of discrete cellular populations and subpopulations that represent “cell types.” Once clusters have been identified, it is possible to identify combinations of genes that are specifically enriched in particular clusters. Many of these marker genes have been previously described for different cell types in the cortex, however it is also possible to discover novel markers. Second, scRNA-seq experiments measure the transcriptome of individual cells that may be in the process of transitioning through various developmental states (e.g., from intermediate progenitor to early neuron). Since not all cells are in the exact same state of differentiation, the transcriptome of each cell can be thought as a representation of a single point of a developmental timeline. By linking the transcriptome from multiple individual cells following a similar developmental program, it is possible to determine the relative position of each cell across the reconstructed trajectory. In this way, entire differentiation trajectories can be reconstructed based on the overlap of gene expression landscapes for cells in the developmental continuum (Haghverdi et al., 2015; Setty et al., 2016; Qiu et al., 2017). This so-called “pseudotime” ordering provides information about the dynamics of gene expression and the establishment of cellular identity in a developing tissue.

**Figure 1:**
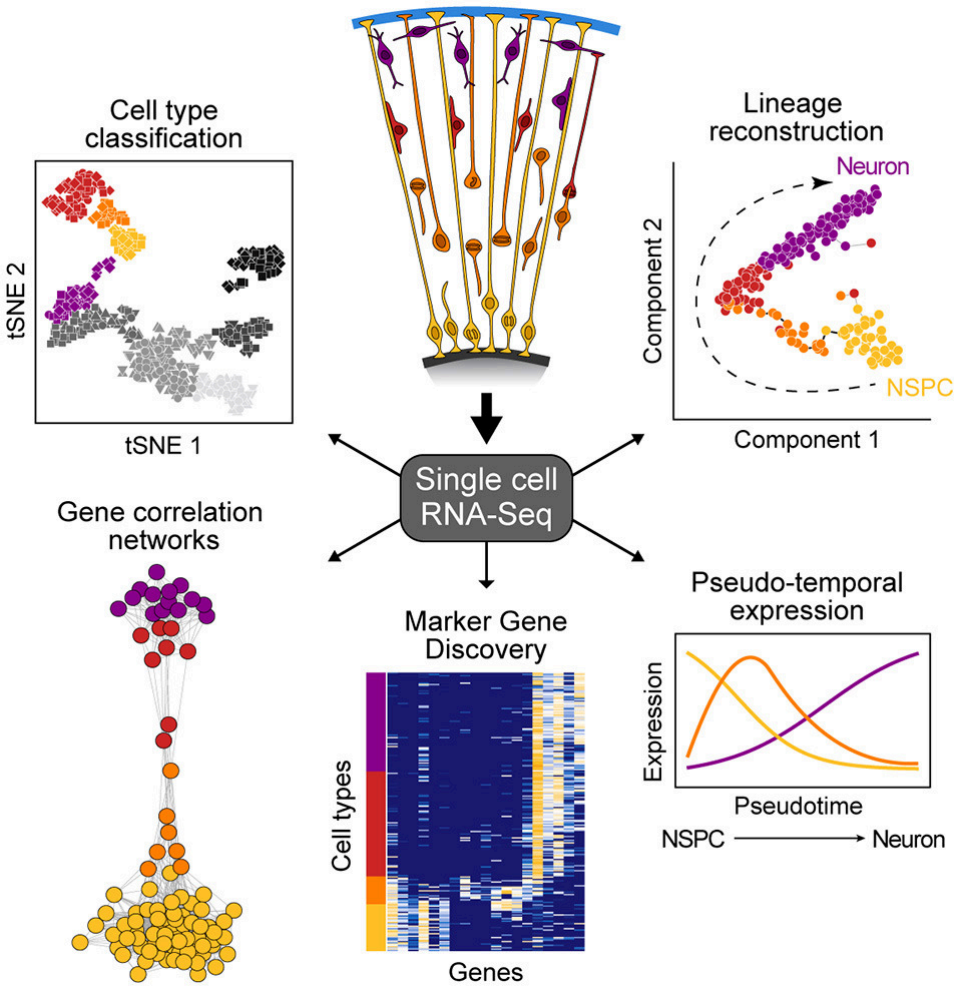
Overview of single-cell RNA-seq data analysis. scRNA-seq allows for the study of heterogeneous tissue by avoiding the need to isolate specific cell types inherent to bulk functional genomic approaches. Such data can be used to classify cell types, elucidate gene networks, identify key genetic markers of cell populations, and delineate differentiation trajectories that describe the transcriptional changes cells undergo during development.

Recently, these methods have been applied to the developing human and mouse cortex, which has led to remarkable progress in understanding the molecular signatures that define cell states within these tissues (Camp et al., 2015; Pollen et al., 2015; Nowakowski et al., 2017). In addition, new protocols have been developed that generate three-dimensional human cortical tissue from induced pluripotent stem cells (Lancaster et al., 2013; Sasai, 2013; Qian et al., 2016; Birey et al., 2017). scRNA-seq dissections of engineered cortical tissues have revealed that the cell type-specific gene expression landscapes are very similar to fetal counterparts (Camp et al., 2015), making these excellent systems to study the genetic mechanisms underlying human-specific cortex development. These published studies have been essential to disentangle some of the developmental processes in cortex development and they provide a rich data resource for further studies. Here we briefly describe the data from four of these publications, and we compile the data into a browseable application called ShinyCortex (https://bioinf.eva.mpg.de/shiny/sample-apps/ShinyCortex/) that biologists can use to explore the gene expression patterns from these publications.

Pollen et al. used scRNA-seq (Fluidigm C1 microfluidic platform, SMART-seq, full length) to identify the molecular signatures that mark radial glia cells located in the outer sub-ventricular zone (known as outer or basal radial glial, oRG/bRG) (Pollen et al., 2015). The expansion of this particular population of RG cells in humans is thought to underlie the expansion of the neocortex on the human lineage (Lewitus et al., 2013). The authors microdissected the VZ and OSVZ and used scRNA-seq data from each location to classify and identify distinct RG populations (vRG and oRG). Their results shed light on the molecular characteristics that establishes the oRG identity, such as the production of trophic factors and extracellular matrix proteins, and the activation of the STAT3 signaling pathway.

Camp et al. (2015) used scRNA-seq (Fluidigm C1 microfluidic platform, SMART-seq, full length) to compare cerebral organoids to human fetal cortical tissue at 12–13 weeks post-conception. The authors first used scRNA-seq to establish a reference atlas of cell composition, progenitor-to-neuron differentiation trajectory, and gene expression networks in the early fetal human cortex at a time point comparable to cerebral organoid development. The authors used a cerebral organoid protocol (Lancaster protocol) (Lancaster and Knoblich, 2014) designed to mimic the early stages of brain development, which allows the organoid to self-organize into cerebral tissue containing multiple brain regions. The authors microdissected individual cortical regions and performed scRNA-seq on the dissociated tissue. The authors directly compared the fetal and organoid cortical cells and found that cellular subtypes in organoids and fetal tissue follow very similar gene expression programs.

Quadrato et al. (2017) used droplet microfluidics (Drop-seq, 3′ end counting, UMIs) to profile more than 80,000 individual cells derived from 31 brain organoids based on a modification of the Lancaster protocol. The organoids were analyzed at different times points (3–6 months), which revealed that neurons mature into potentially active neural networks within the organoid. Notably, the organoids contain multiple brain regions, and here we isolated the cortical cell clusters for compilation into the ShinyCortex application.

Birey et al. used signaling molecules to direct the development of 3D structures called spheroids that resemble two regions of the forebrain (the ventral forebrain and dorsal pallium). Spheroid fusion led to the formation of forebrain-like organoids and interneurons migrated from the ventral to the dorsal region, providing information on brain-region interactions. These authors also performed single-cell transcriptomics (BD Resolve microwell platform, 3' end counting, UMIs) on both ventral and dorsal forebrain spheroids individually and we compiled the dorsal forebrain data into the ShinyCortex application.

Telley et al. analyzed the transcriptome of single cells (Fluidigm C1 microfluidic platform, SMART-SEQ, full length) isolated from multiple time points from the developing mouse neocortex (Telley et al., 2016). The authors were able to bring temporal resolution into their scRNA-seq data by incorporating fluorescent tagging of newborn cells in the ventricular zone. This enabled the authors to identify early transcriptional waves that instruct the sequence and pace of neuronal differentiation events in the mouse cortex. We added this data set to the ShinyCortex application to enable a comparison between human and mouse cell types in the developing neocortex.

Even though the raw sequencing data and processed gene expression matrices for these studies are publicly available online, it can be difficult for researchers to access and analyze this data. ShinyCortex consolidates the processed data into a web accessible Shiny application constructed using the R programming language and based on Plotly, which creates and displays interactive plots that are relatively intuitive and easy to manipulate (https://bioinf.eva.mpg.de/shiny/sample-apps/ShinyCortex/). ShinyCortex can be used to visualize the transcript level of any gene of interest as a function of cell types or over pseudotime, and it allows the user to explore the expression correlations between genes. Generally, the user of ShinyCortex first chooses the dataset that he/she wants to explore (i.e., Pollen, Camp fetal data, Camp organoid data, Quadrato, Birey, Telley). The application is then divided into four sections. The first panel plots the distribution of gene expression values of any gene of interest according to the cell type assignment from each study, and options include box, violin, or scatter plots (Figure 2A). The second panel identifies for a given gene of interest the top correlated or anti-correlated genes and visualizes the correlation coefficients between the genes in the form of correlograms (Figure 2B). Also, it allows the user to include additional genes that may be relevant for certain cellular processes, but are not among the highest correlated genes. The third section uses a generalized additive model to show how gene expression is changing according to pseudotime (Figure 2C), provided that pseudotime values were determined for individual cells in the chosen study. It allows for the display of multiple genes at the same time with visualization of both raw values and smoothened curves. Finally, the last panel uses a heatmap representation to display the expression of multiple genes of interest in individual cells that are ordered either based on their pseudotime (if available) or their cell type assignment (Figure 2D). All plots can be downloaded, and there are graphical parameters than can be modified in each individual plot, such as color palette and panel size. We believe that ShinyCortex will help the corticogenesis community access and explore cortical scRNA-seq datasets, which can lead to the identification of cell state-specific genes for functional analysis.

**Figure 2:**
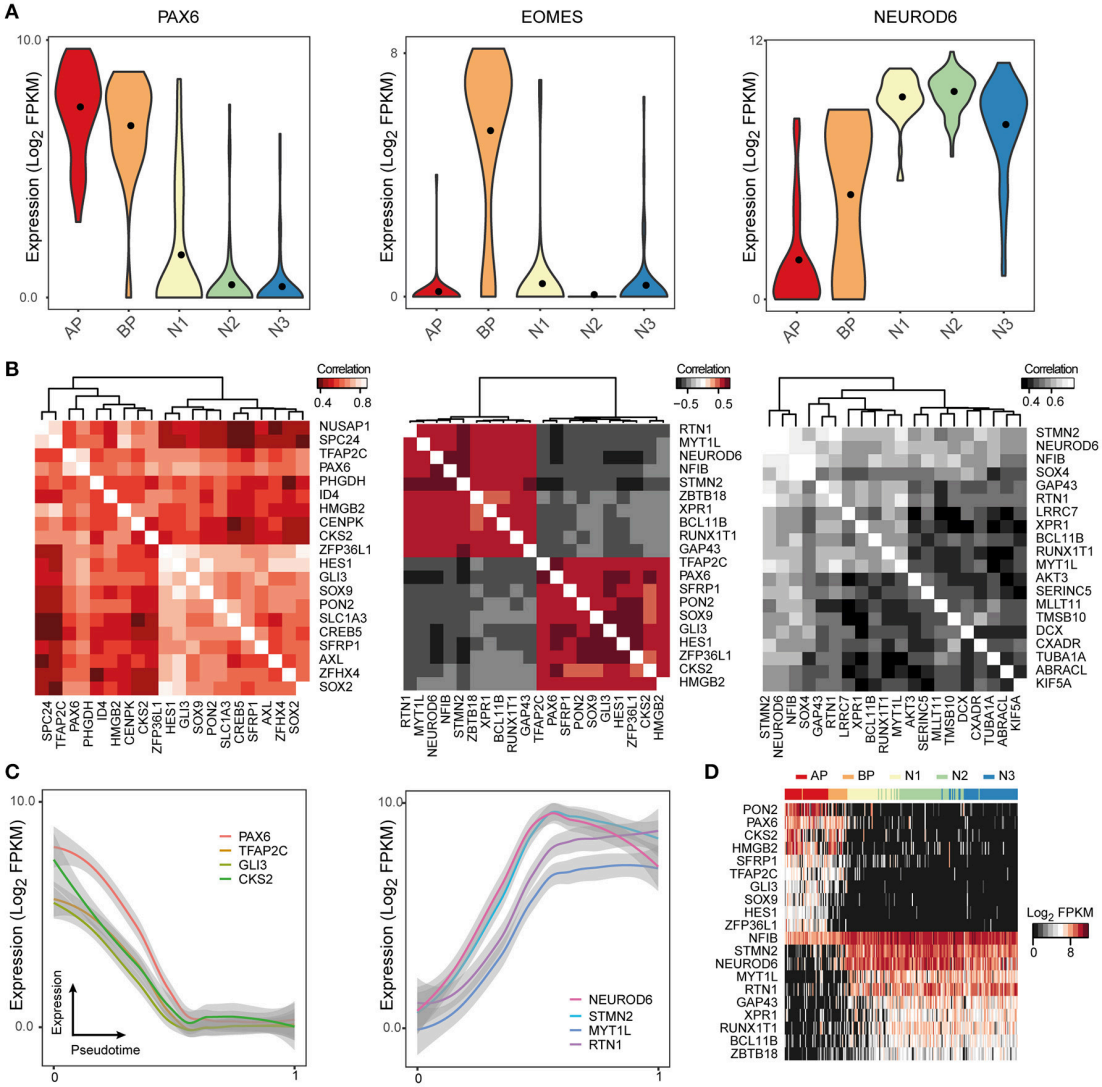
ShinyCortex allows for interactive visualization of neocortex scRNA-seq data. The application can be used to plot and explore various analyses from each dataset starting from a user-defined gene. Representative visualizations are shown here for the fetal neocortex data from Camp et al. **(A)** Violin plots are dynamically generated showing the distribution of gene expression across cell types. **(B)** Heatmaps portray the highest correlated and anticorrelated genes to the selected gene of interest. **(C)** Line plots display the expression profile of genes of interest across developmental pseudotime with level of confidence interval shown (0.95 by default). **(D)** Heatmaps show the expression of selected genes across all cells of a given dataset.

## Methods

All data was obtained through their different data repositories as described in the original papers and was used without any additional processing or filtering. Cell type classification (and pseudo-time if applicable) for each dataset was obtained directly through the authors. Person's correlation coefficients between genes were calculated with r base. All interactive plots were generated with plotly and ggplot2 R packages. Correlation heatmaps were generated with the gplot R package. The pseudotime plots use a generalized additive model (GAM) implemented with the mgcv R package and level of confidence interval (0.95 by default). Pseudotime heatmaps were produced with the stats R package.

## Author contributions

All authors listed have made a substantial, direct and intellectual contribution to the work, and approved it for publication.

### Conflict of interest statement

The handling Editor declared a shared affiliation, though no other collaboration, with one of the authors BT. The other authors declare that the research was conducted in the absence of any commercial or financial relationships that could be construed as a potential conflict of interest.
